# JNK Signalling Controls Remodelling of the Segment Boundary through Cell Reprogramming during *Drosophila* Morphogenesis

**DOI:** 10.1371/journal.pbio.1000390

**Published:** 2010-06-08

**Authors:** Melanie Gettings, Fanny Serman, Raphaël Rousset, Patrizia Bagnerini, Luis Almeida, Stéphane Noselli

**Affiliations:** 1 Institute of Developmental Biology and Cancer, University of Nice, CNRS, Nice, France; 2 DIPTEM, Università degli Studi di Genova, Genova, Italy; 3 Laboratoire JA Dieudonné, University of Nice, CNRS, Nice, France; University of Zurich, Switzerland

## Abstract

Segments are fundamental units in animal development which are made of distinct cell lineages separated by boundaries. Although boundaries show limited plasticity during their formation for sharpening, cell lineages make compartments that become tightly restricted as development goes on. Here, we characterize a unique case of breaking of the segment boundary in late drosophila embryos. During dorsal closure, specific cells from anterior compartments cross the segment boundary and enter the adjacent posterior compartments. This cell mixing behaviour is driven by an anterior-to-posterior reprogramming mechanism involving de novo expression of the homeodomain protein Engrailed. Mixing is accompanied by stereotyped local cell intercalation, converting the segment boundary into a relaxation compartment important for tension-release during morphogenesis. This process of lineage switching and cell remodelling is controlled by JNK signalling. Our results reveal plasticity of segment boundaries during late morphogenesis and a role for JNK-dependent developmental reprogramming in this process.

## Abstract 2

Reprogramming of a specific group of *Drosophila* epidermal cells allows the mixing of normally segregated populations and the release of mechanical tension that arises during morphogenesis.

## Author Summary

Multicellular organisms are assembled from different cell types, each following a particular fate depending on their history and location. During development, cells are organized into compartments, which are essential for the correct formation of organs. Within the compartments, cells follow two general rules: (i) cells that have acquired a given fate cannot change their differentiation state and (ii) cells from one compartment stay together and never mix with cells from other compartments. In this work, we identified a group of unique cells in *Drosophila melanogaster* embryos called mixer cells which move from one compartment of the epidermis to another, breaking the compartment boundary rule. Our data show that this unique behaviour depends on the nuclear reprogramming of the mixer cells, which change their fate and acquire the identity of the destination compartment de novo. We show that the shift in identity and compartment mixing are due to the expression of a single gene (*Engrailed*), under the control of JNK, a signalling pathway that is conserved across species. Interestingly, this process of reprogramming and mixing provides a mechanism of tension relaxation to the tissue during morphogenesis that allows dorsal closure of the *Drosophila* embryo (an event that resembles wound healing). This work reveals a novel model of cell plasticity that is amenable to genetic study, with potential application in the field of regenerative medicine.

## Introduction

Patterning of tissue progenitors through specific gene expression precedes tissue morphogenesis. Once cells are committed to a particular lineage, they generally keep to it throughout development. Nonetheless, plasticity of segmental lineages is commonly observed during the stages of boundary sharpening, like for example during Drosophila segmentation [1],[2],[3],[4] and rhombomere formation in the vertebrate hindbrain [5],[6],[7],[8],[9],[10]. In contrast, during later development, reprogramming of patterned cells is mostly associated with pathological conditions (e.g. regeneration) [11] or experimental procedures (e.g. cloning, grafting, or overexpression of selector genes) [12]. Rare cases of fate switching have nonetheless been reported during somitogenesis and hindbrain segmentation in the chick embryo [5],[13],[14] and during *Caenorhabditis elegans* embryogenesis [15]. Still, whether patterning can be re-adjusted during late tissue morphogenesis remains elusive.

Dorsal closure in Drosophila embryos is a powerful model of epithelial morphogenesis and wound-healing [16],[17],[18]. It proceeds through cell stretching and a zipping mechanism that lead to the convergence and suture of the lateral leading edges (LE) at the dorsal midline (see Video S1). This cell movement is believed to be collective and uniform. By looking at dorsal closure in live Drosophila embryos, we reveal a highly stereotyped pattern of cell reprogramming and intercalation, resulting in the remodelling of segment boundaries during late epithelial morphogenesis.

## Results/Discussion

### Cell Mixing and Intercalation at the Segment Boundaries during Dorsal Closure

**Figure 1:**
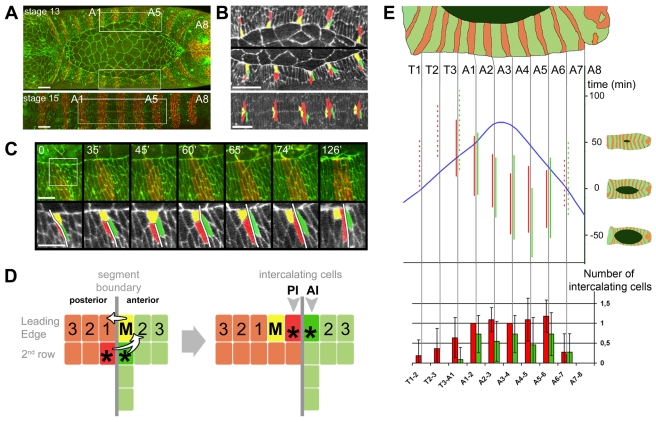
Cell mixing and intercalation at the segment boundaries during dorsal closure. (A) Still confocal images from stage 13 and stage 15 live embryos expressing ubiquitous βCatenin-GFP (green) and Actin-CFP (red) specifically in the posterior compartments (*en>actin-CFP*). (B) High magnifications of bracketed regions in (A) (A1–A5 segments) showing spatial organization of Mixer cells (yellow) and intercalating cells (green and red). (C) Still images from Video S2 showing the dynamics of mixing and intercalation at the A3-A4 boundary (white line). (D) Scheme of cell mixing and intercalation at the segment boundary (M, Mixer cell; AI, anterior intercalating cell; PI, posterior intercalating cell). (E) Upper part: timing and extent of anterior and posterior intercalations (vertical green and red lines, respectively) relative to the time of segment closure (blue curve) (*n* = 5 independent embryos staged with time 0 corresponding to the closure of segment A7, means ± s.d. are given in Table S1). Continuous line: cell intercalates in more than 50% of cases. Dotted line: probability <50% (according to values found in bottom part). Bottom part: final number of intercalating cells at each segment boundary (data are mean ± s.d. with *n* = 11). Scale bars: 20 µm in (A) and (B), 10 µm in (C).

Tracking of the dorsal ectoderm cells using confocal live imaging revealed several unexpected cell rearrangements taking place within the leading edge (Figure 1A–D and Video S2). First, we observed that in abdominal segments, one cell from each anterior compartment mixes with the posterior compartment by the end of dorsal closure. We designate these versatile cells the mixer cells (MCs; yellow in Figure 1B–D). These cells have been noticed recently and have been qualified as an aberration in patterning [19]. Second, we show that two cells from the ventral ectoderm intercalate into the leading edge, posterior to each MC (Figure 1C,D). The two intercalating cells, one from the anterior compartment (anterior intercalating, AI; green in Figure 1B–D) and the other from the posterior compartment (posterior intercalating, PI; red in Figure 1B–D), thus establish new segment boundaries dorsally (Figure 1D and Video S3). This striking pattern of remodelling is spatially and temporally regulated along the leading edge, with a degree of fluctuation, from embryo-to-embryo, in the timing and number of intercalating cells (Figure 1E).

**Figure 2:**
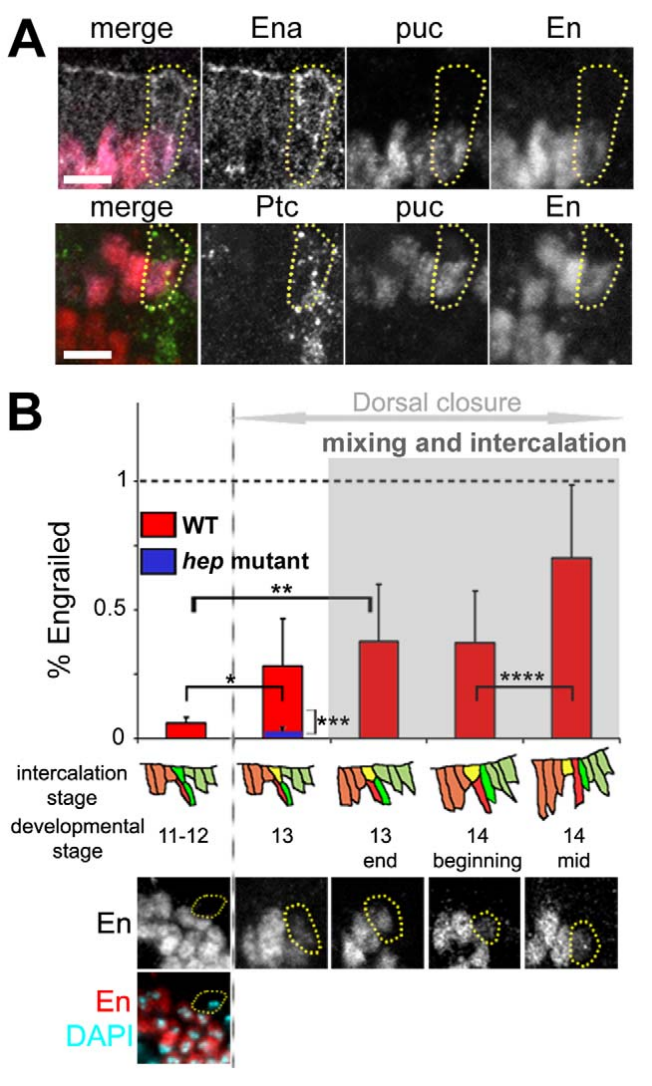
Mixer cells express Engrailed de novo. (A) The mixer cell (yellow dotted circle) expresses high levels of Ena (white, top panel), Ptc (green, bottom panel), and β-Galactosidase (*puc-lacZ*) (purple), indicating it is the dorsal most anterior groove cell. Despite its anterior identity, the mixer cell starts expressing the posterior marker En (red) before shifting from the anterior to posterior compartment (top and bottom panels). (B) Time-course of En expression in the mixer cell. Graph shows relative amounts of En in the mixer cell compared to bona fide posterior En cells at different intercalation stages in wild type (red) and *hep* mutant embryos (blue). Examples of images used for quantification in wild type embryos are shown below the graph. Data are means ± s.d. For WT *n* = 9, 6,12,14,12 cells; for *hep* mutant *n* = 6 cells (* *p* = 0.026, ** *p*<10^−4^, *** *p* = 0.015, **** *p* = 0.0015). Scale bars: 5 µm.

To investigate the mixing mechanism, we analysed the origin and identity of the MCs during dorsal closure. Originally, the MCs occupy the dorsal-anterior corner of each anterior compartment (Figure 1C,D). They are clearly identifiable as part of a single row of cells, known as the groove cells, which form a morphological furrow that marks each segment border, perpendicularly to the leading edge [20],[21]. Like other groove cells, the MCs express higher levels of the actin anti-capping protein Enabled (Ena) (Figure 2A; Figure S1A). The anterior nature of the MCs was confirmed by looking at endogenous Patched (Ptc) expression, which is indeed present throughout the process of cell mixing (Figure 2A; Figure S1B). Thus, both its initial position as well as the expression of Ena, Ptc, and of compartment specific drivers (*ptc-gal4* positive and *en-lacZ* negative; see Figure S1C) show that the MC is the dorsal-most anterior groove cell.

### De Novo Expression of Engrailed in the MC Induces Its Shifting to the Posterior Compartment

The MC behaviour challenges the compartment boundary rule stating that cells from different compartments cannot mix due to different cell affinities that sort them out [2],[3],[4],[10],[22],[23]. One possible explanation for the violation of this law is that the MCs may be re-programmed to acquire posterior identity. Strikingly, the analysis of endogenous protein levels revealed that the MCs start expressing the selector protein Engrailed (En) [24] prior to their shifting towards the posterior compartment (Figure 2A,B; Figure S1 data). The profile of En accumulation in the MCs is distinct from bona fide posterior En-expressing cells present in the neighbouring posterior compartment (Figure 2B; Figure S2), suggesting that En expression in the MCs is controlled by a different mechanism. Double staining for endogenous En and Ptc shows that the MCs express both markers (Figure 2A; Figure S1B), Ptc first then both, which supports the idea that the MCs were originally anterior cells that subsequently acquired posterior identity. Consistent with previous work showing that ectopic expression of En in anterior cells is sufficient to determine posterior-type cells [25],[26], these results suggest that the MCs undergo anterior-to-posterior reprogramming through de novo expression of the En posterior determinant, thus favouring their mixing into the posterior compartment.

**Figure 3:**
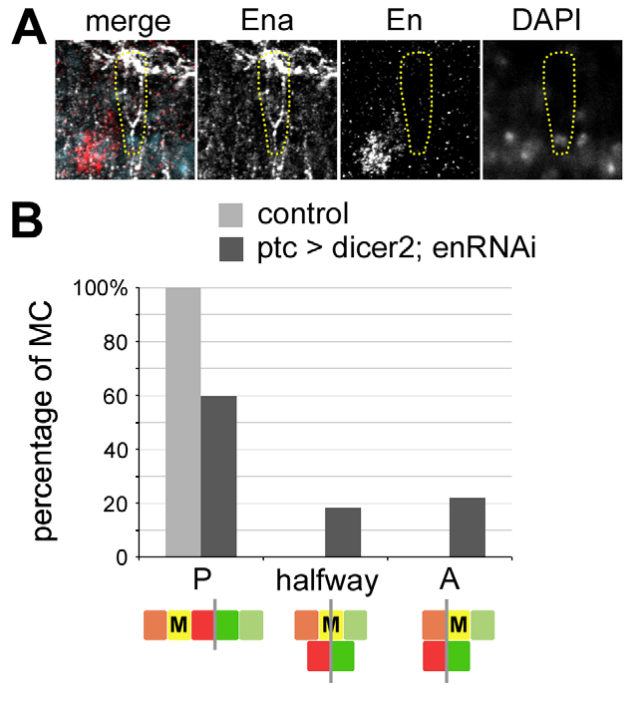
De novo expression of En in the mixer cell controls mixing. (A) *ptc>dicer2; enRNAi* embryos show a loss of En expression in the MC (yellow dotted circle). Ena, white; En, red; DAPI, turquoise. (B) Cell mixing is reduced in *ptc>dicer2; enRNAi* embryos compared to control embryos (*puc-lacZ/+*). The presence of MCs at the end of dorsal closure in the posterior compartment (i.e. mixing; P), in the anterior compartment (i.e. no mixing; A), or in between the two compartments (i.e. incomplete mixing; halfway) was evaluated in the A2–A4 segments and expressed as percentages of MC in the three positions. *n* = 76 segments for 16 control embryos; *n* = 82 segments for 17 *en-RNAi* embryos. The colour codes of the diagrams are the same as in Figure 1D.

To demonstrate a direct role of En in MC formation, we inhibited its function in the anterior compartment by inducing *en* RNAi using the *ptc-gal4* driver. These embryos showed a decrease of En expression in the MC (Figure 3A). In addition, they exhibited a significant number of segments (40%) with aberrant cell mixing, i.e. with partial or no mixing at all (Figure 3B). These results indicate that de novo expression of En in the MCs is essential for their reprogramming and mixing behaviour.

### JNK Signalling Controls En Expression and Cell Mixing

**Figure 4:**
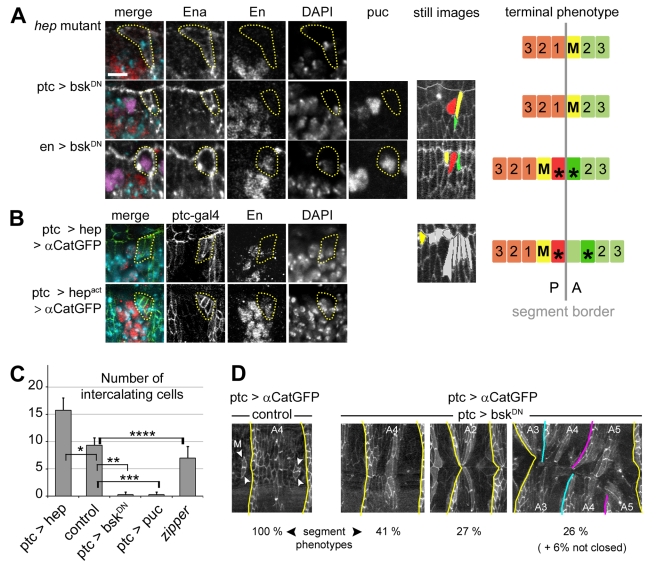
JNK signalling controls En expression in mixer cells. (A, B) Pattern of cell mixing and intercalation in JNK loss and gain of function embryos. A summary of the terminal phenotypes is shown on the right panel. Ena, white; Ptc, green; DAPI, turquoise; β-Galactosidase (*puc-lacZ*), purple; En, red. Still images from live embryos show terminal phenotypes using the colour code as in Figure 1D: mixer (M) cell, yellow; anterior and posterior intercalating cells, green and red, respectively; unidentified intercalating cells, gray. (A) Cell mixing, cell intercalation, and En expression in the mixer cell (yellow dotted circle) are blocked in *hep* mutants and when JNK signalling is selectively down-regulated in the anterior compartment (*ptc>bsk^DN^*). Quantification of En expression in *hep* mutants is shown in Figure 2B. Mixing, intercalation, and En expression are normal when JNK signalling is down-regulated in the posterior compartment (*en>bsk^DN^*). (B) Up-regulation of the JNK pathway in the anterior compartment (*ptc>hep* or *ptc>hep^act^*) induces ectopic mixer cells expressing En in the groove. (C) Total number of intercalating cells per leading edge from wild type (*n* = 6), *ptc>hep* (*n* = 8), *ptc>bsk^DN^* (*n* = 6), *ptc>puc* (*n* = 10), and zipper mutant (*n* = 5) backgrounds. Data are means ± s.d. (* *p* = 0.0015, ** *p* = 0.0026, *** *p*<0.001, **** *p* = 0.06). (D) Segment mismatching in *ptc>αcat-GFP, bsk^DN^* embryos. Percentages of defects are given for segments A1 to A6 (*n* = 144 segments of 24 embryos). Scale bars: 5 µm.

The differentiation of the dorsal leading edge, to which MCs belong, is under the control of the conserved JNK pathway. Embryos lacking the activity of the *JNKK/hemipterous (hep)* gene do not express the LE reporter line *puckered-lacZ* (*puc-lacZ*), fail to undergo dorsal closure and die later in development [27]. Interestingly, *JNKK* mutant embryos are completely lacking cell intercalation and MC shifting (Figure 4A). The expression of Ptc and Ena is normal in these embryos, showing that the identity of the groove cells is not affected in *JNKK* mutants (Figure 4A; Figure S3A). In contrast, expression of En could not be detected in the MCs (Figure 4A top; Figure S3A), which indicates that JNK signalling is essential for de novo En expression. To distinguish compartment specific activities, a dominant negative form of Drosophila JNK/Basket (Bsk^DN^) was expressed either in the anterior or in the posterior compartment using the *ptc-gal4* or *en-gal4* driver, respectively. The extinction of JNK activity was assessed by the loss of *puc-lacZ* expression (Figure 4A). Embryos expressing Bsk^DN^ in the posterior compartment (*en>bsk^DN^*) showed no phenotype (Figure 4A). In contrast, expression of Bsk^DN^ in the anterior compartment (*ptc> bsk^DN^*) led to the complete absence of MC intercalation, as is observed in *JNKK* mutant embryos (Figure 4A,C; Figure S4 and Video S4). The same result was obtained when blocking JNK signalling through overexpression of the JNK phosphatase Puckered (Puc) (Figure 4C; Figure S3B). Absence of JNK activity in *ptc>bsk^DN^* or *ptc>puc* embryos (but not with the *en-gal4* driver) also led to the abolition of En expression in the MCs (Figure 4A; Figure S3B). Interestingly, although most (87%) *ptc>bsk^DN^* embryos were able to complete dorsal closure, 92% of them showed a high degree of segment mismatching at the dorsal midline (53% of A1–A6 segments showed defects; Figure 4D). This suggests that MC formation and intercalation play a role in segment adjustment at the time of suture, consistent with a previous hypothesis [19]. In contrast, matching was normal in *en>bsk^DN^* embryos. Together, these results indicate that JNK signalling is essential in the anterior compartment, most likely in the MCs, to promote anterior-to-posterior reprogramming through de novo expression of En, compartment mixing, and segment adjustment.

In order to address the effect of excess JNK activity in the process, we ectopically expressed either a wild type (Hep) or an activated form of DJNKK (Hep^act^) in the anterior compartment using the *ptc-gal4* driver. These gain-of-function conditions induced a dramatic increase in the number of intercalated cells and the formation of ectopic MCs at the segment boundaries (Figure 4B,C; Figures S3C, S5, and Video S5). These ectopic MCs express Ptc, Ena, and En like normal MCs. These results show that more lateral groove cells are competent for reprogramming, but they are restricted by the field of JNK activity in the leading edge.

### Wingless Inhibits Groove and MC Formation

**Figure 5:**
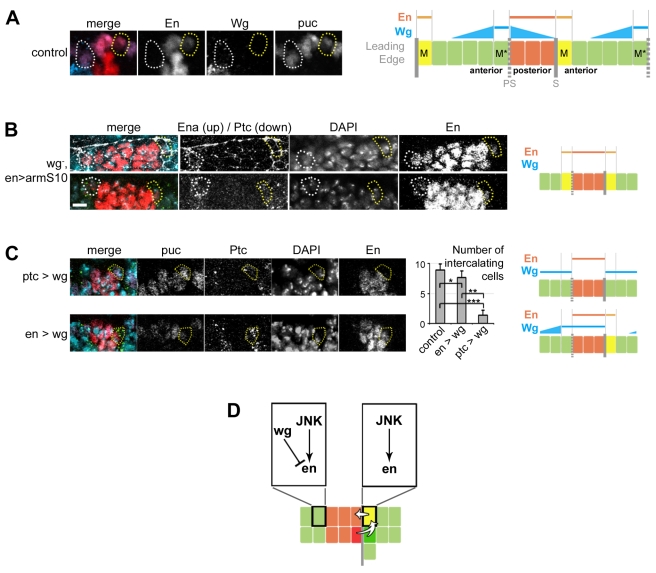
Wg inhibits groove and Mixer cell formation at the parasegment boundaries. (A) Leading edge expression of En and Wg in stage 13 embryos; mixer cell (M), yellow dotted circles; mixer mirror cell (M*), white dotted circles; En, red; Wg, turquoise; β-Galactosidase (*puc-lacZ*), purple. (B) Specific loss of *wg* signalling induces ectopic mixer cell formation at the parasegment boundary as seen by expression of Ena (white, top) and En (red, top and bottom); Ptc (green, bottom); DAPI is turquoise. (C) Overexpression of Wg in the mixer cell (*ptc>wg*, top panel) inhibits anterior-to-posterior reprogramming as seen by the absence of En in the mixer cell, leading to the absence of the mixing (see Video S6). Overexpression of *wg* in the posterior compartment (*en>wg*, bottom panel) has no effect on reprogramming and mixing. The histogram shows the total number of intercalating cells for control (*n* = 8), *ptc>wg* (*n* = 8), and *en>wg* (*n* = 6) embryos. Data are means ± s.d. (* *p* = 0.06, ** *p* = 0.0012, *** *p* = 0.0005). β-Galactosidase (*puc-lacZ*), purple; Ptc, green; DAPI, turquoise; En, red. (A–C) (right panels) Scheme of the phenotype and expression patterns of Wg and En; PS is for parasegment boundary and S for segment boundary. (D) Model of JNK induced reprogramming at the segment boundary and Wg inhibition at the parasegment boundary. Scale bars: 5 µm.

Each MC has a mirror-image counterpart at the LE parasegment (PS) boundary (MC* in Figure 5A) that never develops into a MC. Interestingly, the asymmetry of the MC pattern correlates with Wingless (Wg) activity across the segment [28] and the presence of the groove at the segment boundary (Figure 5A) [20],[21]. In addition, in JNK gain-of-function embryos, extra MCs only appear along the segment boundary (Figure 4B), suggesting that only groove cells can differentiate into MCs. To test this hypothesis, we made use of specific *wg* mutant embryos in which an ectopic groove is formed at the PS boundary [20]. In this context, MCs* were transformed into ectopic MCs at the PS boundary (Figure 5B). Like genuine MCs, transformed MCs* express Ptc, Ena, and most importantly En, which suggests that Wg suppresses the MC pathway at the PS boundary. To test whether Wg itself can repress MC formation, Wg was expressed ectopically in the MCs (*ptc>wg*) where it is not normally active [28]. This blocks MC reprogramming and cell remodelling (Figure 5C; Video S6). Consistently, En expression is no longer detected in MCs. These results indicate that Wg has a non-permissive function at the PS boundary through the blocking of groove cell differentiation, thus restricting the MC pathway to the segment boundary (Figure 5D). Therefore, only dorsal groove cells are competent for MC formation (Figure 5D).

### Local Tissue Tension Modifies the Dynamics of Cell Intercalation

**Figure 6:**
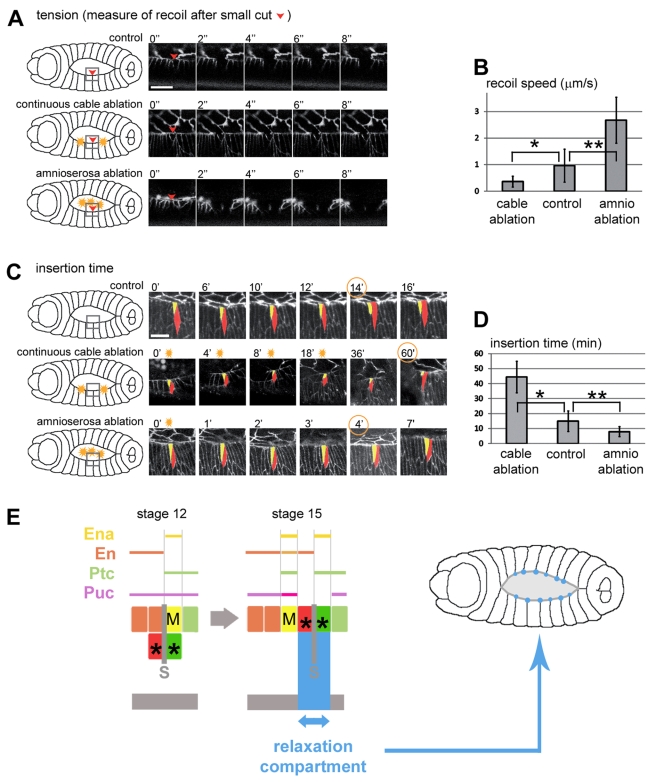
Local tissue tension modifies the dynamics of cell intercalation. (A) Still confocal images showing cable recoil following laser ablation (from stage 14 live embryos expressing βCatenin-GFP, in gray). Single cell ablations are done in the mixer cell of a central segment (red arrowheads). The upper panel shows ablation in a control embryo cut once. The middle panel shows the recoil following a double ablation targeting adjacent segments (yellow sparkles in scheme on the left side). The bottom panel shows the recoil following the ablation of half of the amnioserosa (see scheme on the left side). (B) Indirect measurements of local tension at the segment boundary (recoil speeds) in control embryos, embryos with cable ablation, and embryos with amnioserosa ablation (*n* = 6, 3, and 6, respectively). Data are means ± s.d. (* *p* = 0.167, ** *p* = 0.048). (C) Still confocal images showing the timing of insertion in the leading edge of a control embryo (upper panel), and embryos with continuous cable ablation (middle panel) or with amnioserosa ablation (bottom panel). Mixer cell, yellow; posterior intercalating cell, red. (D) Timing of the final phase of intercalation (leading edge insertion) in all three conditions (control, *n* = 22; cable ablation, *n* = 2; amnioserosa ablation, *n* = 11). Data are means ± s.d. (* *p* = 0.032, ** *p* = 0.085). (E) Scheme showing the relaxation (blue arrow) at the segment boundary as a result of cell rearrangements (left panel) and the pattern and variability of relaxation from segment to segment (blue circles of variable sizes, right panel). Scale bars: 10 µm in (A) and (C).

Dorsal closure is characterised by dramatic cell elongation (3-fold in the DV axis) accompanied by the formation of a LE supracellular actin cable and amnioserosa contraction, all of which contribute to tissue tension (see Video S1) [29]–[33]. To test the effect of tension on the intercalation process, we applied laser ablation to live embryos expressing βCatenin-GFP. The tension in tested segments was assessed in three conditions (control, amnioserosa ablation, and cable ablation) by measuring the ectoderm recoil after a single cell ablation at the leading edge (Figure 6B). Increase in LE tension was induced by ablation of the pulling amnioserosa, while its release was induced through a double ablation of the actin cable on each side of a test segment (Figure 6A). We next compared the dynamics of LE insertion in the controls and in embryos mechanically challenged by laser. In control embryos, the PI cell (red in Figure 6C) takes, on average, 14 min to complete insertion in the leading edge. This time increases dramatically when tension is reduced in the cable (cable ablation condition; 60 min, Figure 6C middle panel, 6D), while it is shortened (4 min) in conditions of higher tension generated by amnioserosa ablation (Figure 6C bottom panel, 5D). These data show that the dynamics of intercalation depends on local tissue tension and suggest a role of intercalation in tension modulation. Improper tension release along the leading edge, in the absence of cell intercalation, could therefore explain the reduced ability of segments to match with their counterparts, as observed in JNK mutant conditions (Figure 4D). To perturb tension genetically, we analyzed the pattern of intercalation in *zipper* (*zip*, encoding MyoII) mutants, in which a reduced tissue tension has been reported [34]. Interestingly, these embryos show a reduced level of cell intercalation, supporting our model of a link between tissue tension and the rate of intercalation (Figure 4C).

Based on these results, we propose that the MC pathway provides an adaptive response to tissue tension by allowing an increase of cell number in the leading edge. Indeed, one major consequence of boundary remodelling is the addition of intercalating cells (AI and PI), which increases the cellular number of the leading edge by approximately 10% (Figure 1E). The adaptive nature of cell intercalation is reflected by the flexibility in the number (from 0 to 3) of intercalating cells (Figure 1E), which contrasts with the robustness of MC reprogramming assessed by de novo expression of En. In our model, MC formation would weaken the segment boundary (i.e., through a change in cell affinity), making it a preferred site competent for tension-dependent intercalation. Our data and work published by Peralta et al. [35] indicate that the width remains constant on average with only slight oscillations during dorsal closure. Therefore, for constant width the increase in the number of cells implies that each cell is less stretched, thus inducing tension relaxation in individual cells. MC formation and associated local cell intercalation thus provide each segment with a tuneable relaxation compartment, important for tension release during morphogenesis (Figure 6E).

In this study we unravel the mechanism of a unique case of breaching of the segment boundary during late morphogenesis, i.e. post-patterning and post-boundary sharpening. This process is shown to be highly stereotyped and developmentally regulated through JNK signalling. Our data indicate that it takes place through a two-step mechanism, involving first MC formation, which is then followed by cell intercalation. Indeed, de novo expression of En in the dorsal groove cell always precedes intercalation (Figure 2B). Furthermore, we can observe MC formation and mixing without intercalation like in the thoracic segments (Figure 1E), but intercalation was never observed in the absence of MC formation: for example, when MC reprogramming is blocked in *JNK* loss-of-function conditions, no intercalation occurs (Figure 4A). Cell mixing thus takes place through a novel morphogenetic mechanism involving plasticity of the segment boundary and compartment relaxation via patterned intercalation. It would be interesting to see if plasticity of boundaries can be a general mechanism for fine tuning late morphogenesis. Intriguingly, late expression of En in anterior cells has been reported at the anterior-posterior boundary in the wing imaginal disc. But contrary to the MC process, the so-called “S. Blair cells” do not mix with the posterior En-expressing cells [36], and their function remains elusive. It would be interesting to reinvestigate their late behaviour using time lapse approaches [37].

Interestingly, the JNK pathway has been shown to be involved in transdetermination of injured imaginal discs [38], reminiscent of the MC reprogramming described here. Hence, JNK signalling represents a fundamental morphogenetic and cell reprogramming pathway essential for developmental and regenerative sealing. Work on MC boundary violation and reprogramming provides a novel model to understand the molecular basis of cell plasticity.

## Materials and Methods

### Genetics

The following fly lines were used: β*catenin-GFP* (8556), *UAS-h-actin-CFP* (7064), *UAS-myr-RFP* (7119), *UAS-Dαcatenin-GFP, UAS-hep^act^* (9306), *UAS-bsk^DN^* (6409), *zip^1^* (4199), *UAS-lamGFP* (7377) (all from the Bloomington stock center), *UAS-en-RNAi* (VDRC#35697), *ptc-gal4* (gift from N. Perrimon), *en-gal4* (gift from A. Brand; see Figure S6 for *en-gal4* expression pattern in MCs), *puc^E69^* (*puc-lacZ* [39]), *UAS-puc2a* [40], *UAS-wg* [41], *hep^1^*, *hep^r75^* and *UAS-hep4E* [27], *wg^cx4^*, *en-gal4*, and *wg^cx4^, arm^S10^* [20]. The following recombined lines were used for video time-lapse of dorsal closure in various genetic backgrounds (this study): (1) w*; βcatenin-GFP, en-gal4/UAS-h-actin-CFP; (2) w*, ptc-gal4, UAS-Dαcatenin-GFP; (3) w*/UAS-bsk^DN^; ptc-gal4, UAS-Dαcatenin-GFP; (4) w*; βcatenin-GFP, ptc-gal4; *UAS-hep^4E^*; (5) w*; βcatenin-GFP, ptc-gal4/UAS-lacZ; (6) w*/UAS-bsk^DN^; βcatenin-GFP, ptc-gal4; (7) w*; βcatenin-GFP, ptc-gal4; UAS-puc2a; (8) w*; βcatenin-GFP, en-gal4/UAS-lacZ; (9) w*; βcatenin-GFP, en-gal4; UAS-wg; (10) w*; βcatenin-GFP, ptc-gal4;UAS-wg; and (11) w*/UAS-bsk^DN^; βcatenin-GFP, ptc-gal4; UAS-myr-RFP. Removal of late *wg* function was obtained using *wg^cx4^*, *en-gal4*/*UAS- arm^S10^* embryos [20].

### Antibodies, Immunostaining, Imaging

Embryos were dechorionated in 1.6% bleach, fixed for 15 min in heptane and 4% paraformadehyde diluted in PBS (50∶50 mix), devitellinised in heptane and methanol (or chilled 70% ethanol when presence of GFP) (50∶50 mix) for 2 min using a vortex (or incubated at −20°C for 7 min before vortexing when GFP), rinsed 3 times in methanol, then 3 times in ethanol, rehydrated sequentially in ethanol/PBS 0.1% triton solutions (70/30,50/50,30/70, 0/100) for 5 min each time, then blocked in PBS 0.1% triton 1% BSA for a minimum of 2 h at room temperature before applying primary antibodies for overnight incubation at 4°C. Primary antibodies were washed 6×10 min with the blocking solution at room temperature before adding secondary antibodies for a minimum of 2 h at room temperature. Finally, embryos were treated with DAPI (10 µg/ml, Biochemika) for 5 min at room temperature. 6×10 min washing in PBS 0.1% triton preceded mounting in Mowiol® 4-88 Reagent (Calbiochem). Antibodies used: mouse anti-Ena 5G2 (1/500), mouse anti-Ptc apa I (1/50), anti-Wg 4D4 (1/500) (Developmental Studies Hybridoma Bank), rabbit anti-En (1/200; Santa Cruz), chicken anti-β-Galactosidase (1/1000; Genetex), anti-mouse Al488 (1/400; Molecular Probe), anti-rabbit cy5 (1/100), and anti-chicken cy3 (1/400) both from Jackson.

Images were taken with a Zeiss LSM 510 Meta confocal microscope using ×40 1.3 NA or ×63 oil immersion objectives.

### Live Imaging, Laser Ablation, and Image Treatment

Embryos were dechorionated in bleach, then staged and placed dorsal side down on a coverslip. Embryos were then coated with halocarbon oil and covered with a hermetic chamber containing a piece of damp paper for hydration. This mounting system ensures normal development of 95% of embryos. Videos last from 2 to 5 h with stacks of 25 images (thickness from 30 to 40 µm) taken every 5 min.

Image and video assembly was done using ImageJ. Stacks are projected using either a maximal intensity or an average projection. Cell intercalations were analysed by tracking manually each cell with ImageJ. Graphs were made using Microsoft Excel. Video S3 was made using Microsoft PowerPoint and Alcoosoft PPT2Video converter.

Ablations were performed using a two-photon pulsed Spectraphysic's Tsunami laser combined with a Zeiss LSM 510 Meta confocal microscope for imaging. The power was calibrated in each experiment using a test embryo and ablations were performed with the Zeiss “bleach” macro to control the size and timing of each cut. For MC ablation, the actin cable on the dorsal side was targeted, while for amnioserosa ablation, the laser beam was focused on the apical area to destroy adherens junctions and the cytoskeleton in a region of interest of 10–30 micrometers long, parallel to the AP axis. To determine cable tension, we used the classical definition of tension in a purse string as the magnitude of the pulling force exerted by the string [29]. The application of Newton's second law under the conditions of low Reynolds number (viscous fluid) shows that the initial recoil speed of a cable after the cut is proportional to the contribution of the suppressed force, i.e. tension [32],[42].

### Protein Level Quantification in MCs

ImageJ was used to quantify En and β-Galactosidase levels on projections of non-saturated stacks of images. For a given segment, the absolute intensity of En in the MCs was normalised to the average absolute intensities of the bona fide En-expressing cells of the leading edge. An average of these relative intensities was calculated for stages of intercalation as shown in Figure 2. For each embryo, only segments A2, A3, and A4 were considered as they are most representative of mixing and intercalation. Relative intensity in the MCs is the ratio of absolute MC intensity/average of absolute intensities in bona fide En cells.

### Statistical Analysis

All analyses were performed using the Mann-Whitney non-parametric test, which does not assume any condition on the distribution and is adapted to independent experiments and small sample sizes. *p* values were computed using the statistics toolbox from the Matlab software.

## Supporting Information

Figure S1 **Expression of cellular markers in the mixer cells.** (A, B) Pattern of Ena (A), Ptc (B), *puc-lacZ*, and En expression at three different stages of cell mixing: before anterior-to-posterior mixer cell shifting (top panels); after the onset of mixer cell shifting and cell intercalation (middle panels); end of cell mixing and intercalation (bottom panels). Ena, white; Ptc, green; DAPI, turquoise; β-Galactosidase (*puc-lacZ*), purple; En, red. (C) Pattern of endogenous En expression in an embryo expressing the β-Galactosidase under the control of the *en* enhancer (*en–lacZ*) and αCateninGFP under the control of the *ptc-gal4* driver (*ptc>CatGFP*). Note the expression of En in the most anterior *ptc-gal4* expressing cell (i.e. the mixer cell). αCateninGFP, green; β-Galactosidase, turquoise; En, red. Yellow lines in (A–C) outline the segment boundary. The right panel is a scheme of the intercalation stages (mixer cell, yellow; PI, red; AI, green; posterior, orange; anterior, light green). Scale bars: 5 µm.
(3.08 MB TIF)

Figure S2 **Time-course of Engrailed expression in the mixer cells.** Examples of En stainings used for the quantification of the relative amounts of En in the mixer cells compared to neighbouring bona fide En cells. The bottom panel shows a scheme of the intercalation stages (mixer cell, yellow; PI, red; AI, green; posterior, orange; anterior, light green).Ena, white; DAPI, turquoise; β-Galactosidase, purple; En, red. Yellow lines outline the segment boundary. Scale bars: 100 µm in 1st row; others 5 µm.
(1.78 MB TIF)

Figure S3 **JNK activity controls mixer cell formation and En expression.** (A) Expression of endogenous Ptc and En proteins in a *hep^r75^/hep^1^* mutant embryo. Note that in these mutant embryos the mixer cell expresses Ptc but not En in contrast to wild type embryos. Ptc, green; DAPI, turquoise; En, red. Scale bars: 5 µm. (B) Embryos overexpressing the JNK phosphatase Puc either in the anterior (*ptc>puc*) or the posterior compartment (*en>puc*). Overexpression of Puc mimics overexpression of Bsk^DN^ (see Figure 4A). Note the absence of β-Galactosidase staining in the compartment where JNK is downregulated. Ena, white; DAPI, turquoise; β-Galactosidase, purple; En, red. (C) Up-regulation of the JNK pathway in the anterior compartment (genotype: *ptc-gal4, UAS-hep^act^; puc-lacZ*) induces ectopic *puc-lacZ* positive cells and mixer cells expressing both Ptc and En at the segment boundary. Yellow lines outline the segment boundary. Scale bar: 5 µm.
(1.34 MB TIF)

Figure S4 **JNK down-regulation in the anterior compartment inhibits cell intercalation.** Confocal still images from Video S4 showing an embryo expressing Bsk^DN^ in the anterior compartment. Genotype: *ptc-gal4, UAS-bsk^DN^, UAS-RFP; βcatenin-GFP*. βCatenin-GFP, white; RFP, green. Colour code as in Figure 1B. Scale bars: 10 µm.
(2.31 MB TIF)

Figure S5 **JNK overactivation induces ectopic mixer cells and intercalation.** Confocal time-lapse imaging of an embryo expressing the JNKK Hep in the anterior compartment from Video S5. Genotype: *ptc-gal4, UAS-hep; βcatenin-GFP*. βCatenin-GFP, green. Colour code as in Figure 4A. Scale bars: 10 µm.
(2.41 MB TIF)

Figure S6 **Expression pattern of the** ***en-gal4*** **driver.** Still images of an A4 segment of a *βcatenin-GFP,en-gal4 > UAS-lamin-GFP* live embryo, from early stage 15 to stage 16. (A) Normal image. (B) False colouring (red) of the dorsal row of En cells. (C) Increasing the brightness allows the visualization of lamin-GFP weak expression in the mixer cells (red arrows). Scale bars: 10 µm.
(1.93 MB TIF)

Table S1 **Timing and standard deviations of closure and cell intercalation described in** **Figure 1E** **.**
(0.04 MB DOC)

Video S1 **Dorsal closure of a wild type embryo.** Confocal time-lapse images are taken from an embryo expressing ubiquitous αCatenin-GFP (green) and h-Actin-CFP (red) in the posterior compartment. Genotype: *βcatenin-GFP; en-gal4, UAS-h-actin-CFP*.
(6.40 MB MOV)

Video S2 **High magnification of segment boundary remodelling in a wild type embryo.** High magnification of the A3–A4 segment boundary during dorsal closure showing mixer cell shifting and cell intercalations. Confocal time-lapse images are taken from an embryo expressing ubiquitous βCatenin-GFP (green) and Actin-CFP (red) in the posterior compartment. Genotype: *βcatenin-GFP; en-gal4, UAS-actin-CFP*.
(2.81 MB MOV)

Video S3 **Dynamics of cell mixing and intercalations at the segment boundaries.** Summary cartoon showing the spatial and temporal dynamics of mixer cell shifting and cell intercalations at the segment boundaries.
(2.73 MB MOV)

Video S4 **Absence of intercalation in an embryo defective for JNK signalling in the anterior compartment.** Confocal time-lapse imaging of an embryo expressing a dominant negative form of Bsk in the anterior compartment. Genotype: *ptc-gal4, UAS-bsk^DN^; βcatenin-GFP*. βcatenin-GFP, green. Colour code as in Figure 4A. Scale bars: 10 µm.
(0.82 MB MOV)

Video S5 **Excessive intercalations in an embryo overexpressing JNKK in the anterior compartment.** Confocal time-lapse imaging of an embryo overexpressing the JNKK Hep in the anterior compartment. Genotype: *ptc-gal4, UAS-hep, UAS-RFP; βcatenin-GFP*. βCatenin-GFP, white; RFP, green. Colour code as in Figure 4A. Scale bars: 10 µm.
(0.56 MB MOV)

Video S6 **Absence of intercalation in an embryo overexpressing Wg in the anterior compartment.** Confocal time-lapse imaging of an embryo overexpressing Wg in the anterior compartment. Putative most anterior cells of segments A2 to A6 are coloured in green to show that no intercalation occurs in the entire bracketed region. Genotype: *ptc-gal4, UAS-wg; βcatenin-GFP*. βCatenin-GFP, white. Scale bars: 10 µm.
(1.00 MB MOV)

## Abbreviations

AI: anterior intercalating
En: Engrailed
Ena: Enabled
*hep*: *hemipterous*
MC: mixer cell
PI: posterior intercalating
PS: parasegment
Ptc: Patched
Puc: Puckered
*puc-lacZ*: *puckered-lacZ*
Wg: Wingless
