# Supplementary figures and images for: JNK Signalling Controls Remodelling of the Segment Boundary through Cell Reprogramming during Drosophila Morphogenesis
Source: PLoS Biol. 2010 Jun 8;8(6):e1000390. doi: 10.1371/journal.pbio.1000390 (PMC2882433; doi:10.1371/journal.pbio.1000390)

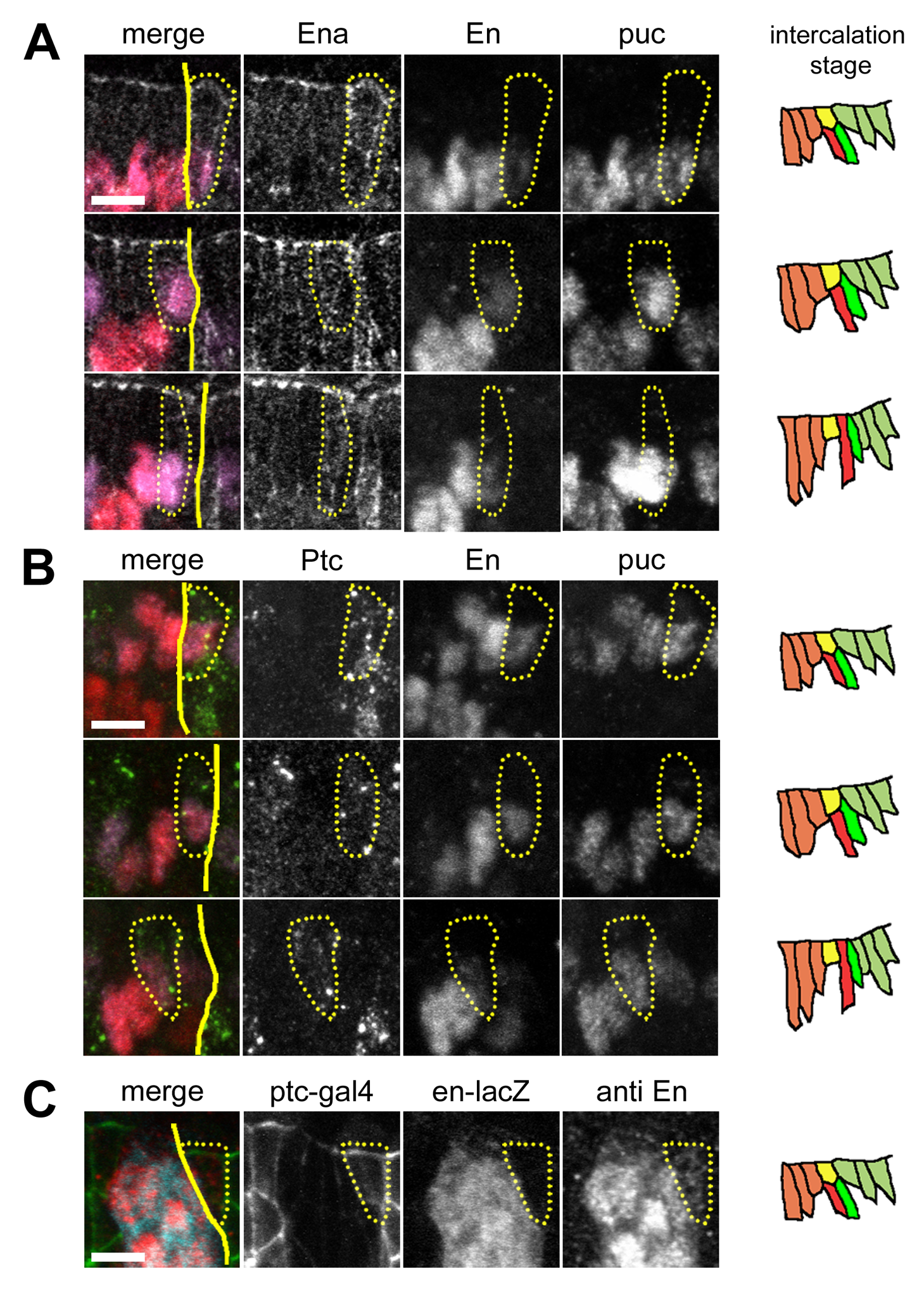

Supplement: Figure S1 — Expression of cellular markers in the mixer cells. (A, B) Pattern of Ena (A), Ptc (B), puc-lacZ, and En expression at three different stages of cell mixing: before anterior-to-posterior mixer cell shifting (top panels); after the onset of mixer cell shifting and cell intercalation (middle panels); end of cell mixing and intercalation (bottom panels). Ena, white; Ptc, green; DAPI, turquoise; β-Galactosidase (puc-lacZ), purple; En, red. (C) Pattern of endogenous En expression in an embryo expressing the β-Galactosidase under the control of the en enhancer (en–lacZ) and αCateninGFP under the control of the ptc-gal4 driver (ptc>CatGFP). Note the expression of En in the most anterior ptc-gal4 expressing cell (i.e. the mixer cell). αCateninGFP, green; β-Galactosidase, turquoise; En, red. Yellow lines in (A–C) outline the segment boundary. The right panel is a scheme of the intercalation stages (mixer cell, yellow; PI, red; AI, green; posterior, orange; anterior, light green). Scale bars: 5 µm. (3.08 MB TIF) [file pbio.1000390.s001.tif]

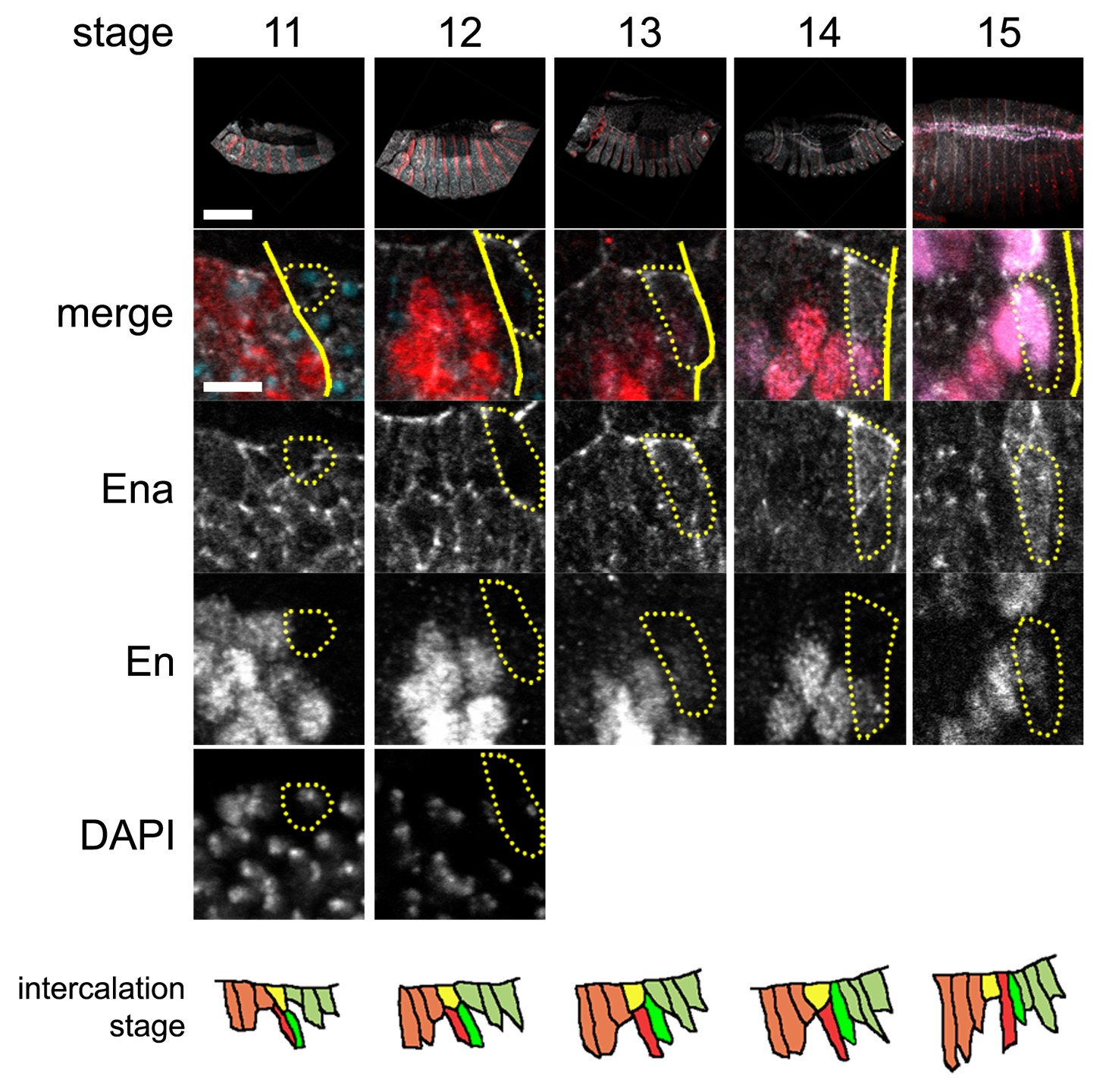

Supplement: Figure S2 — Time-course of Engrailed expression in the mixer cells. Examples of En stainings used for the quantification of the relative amounts of En in the mixer cells compared to neighbouring bona fide En cells. The bottom panel shows a scheme of the intercalation stages (mixer cell, yellow; PI, red; AI, green; posterior, orange; anterior, light green).Ena, white; DAPI, turquoise; β-Galactosidase, purple; En, red. Yellow lines outline the segment boundary. Scale bars: 100 µm in 1st row; others 5 µm. (1.78 MB TIF) [file pbio.1000390.s002.tif]

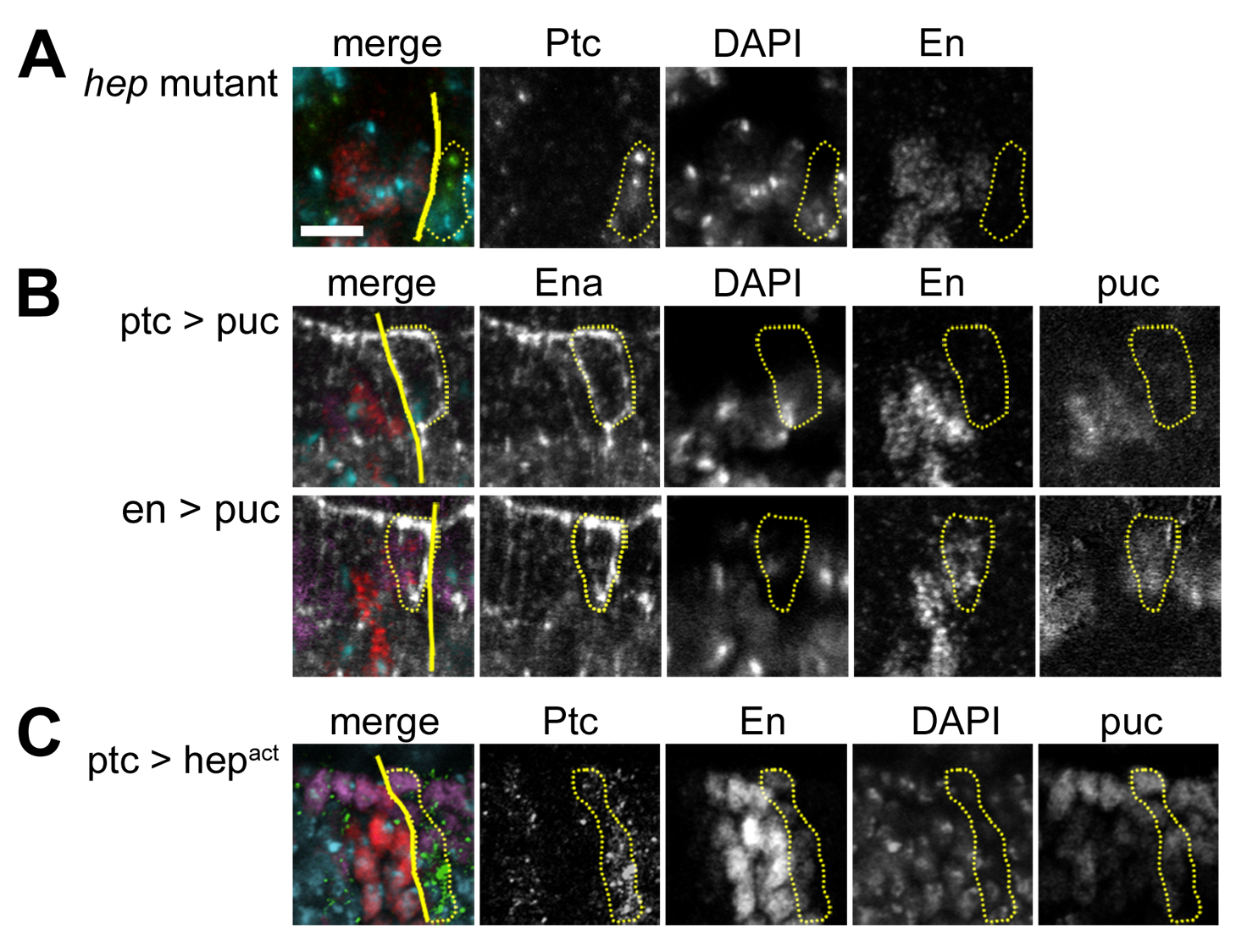

Supplement: Figure S3 — JNK activity controls mixer cell formation and En expression. (A) Expression of endogenous Ptc and En proteins in a hepr75/hep1 mutant embryo. Note that in these mutant embryos the mixer cell expresses Ptc but not En in contrast to wild type embryos. Ptc, green; DAPI, turquoise; En, red. Scale bars: 5 µm. (B) Embryos overexpressing the JNK phosphatase Puc either in the anterior (ptc>puc) or the posterior compartment (en>puc). Overexpression of Puc mimics overexpression of BskDN (see Figure 4A). Note the absence of β-Galactosidase staining in the compartment where JNK is downregulated. Ena, white; DAPI, turquoise; β-Galactosidase, purple; En, red. (C) Up-regulation of the JNK pathway in the anterior compartment (genotype: ptc-gal4, UAS-hepact; puc-lacZ) induces ectopic puc-lacZ positive cells and mixer cells expressing both Ptc and En at the segment boundary. Yellow lines outline the segment boundary. Scale bar: 5 µm. (1.34 MB TIF) [file pbio.1000390.s003.tif]

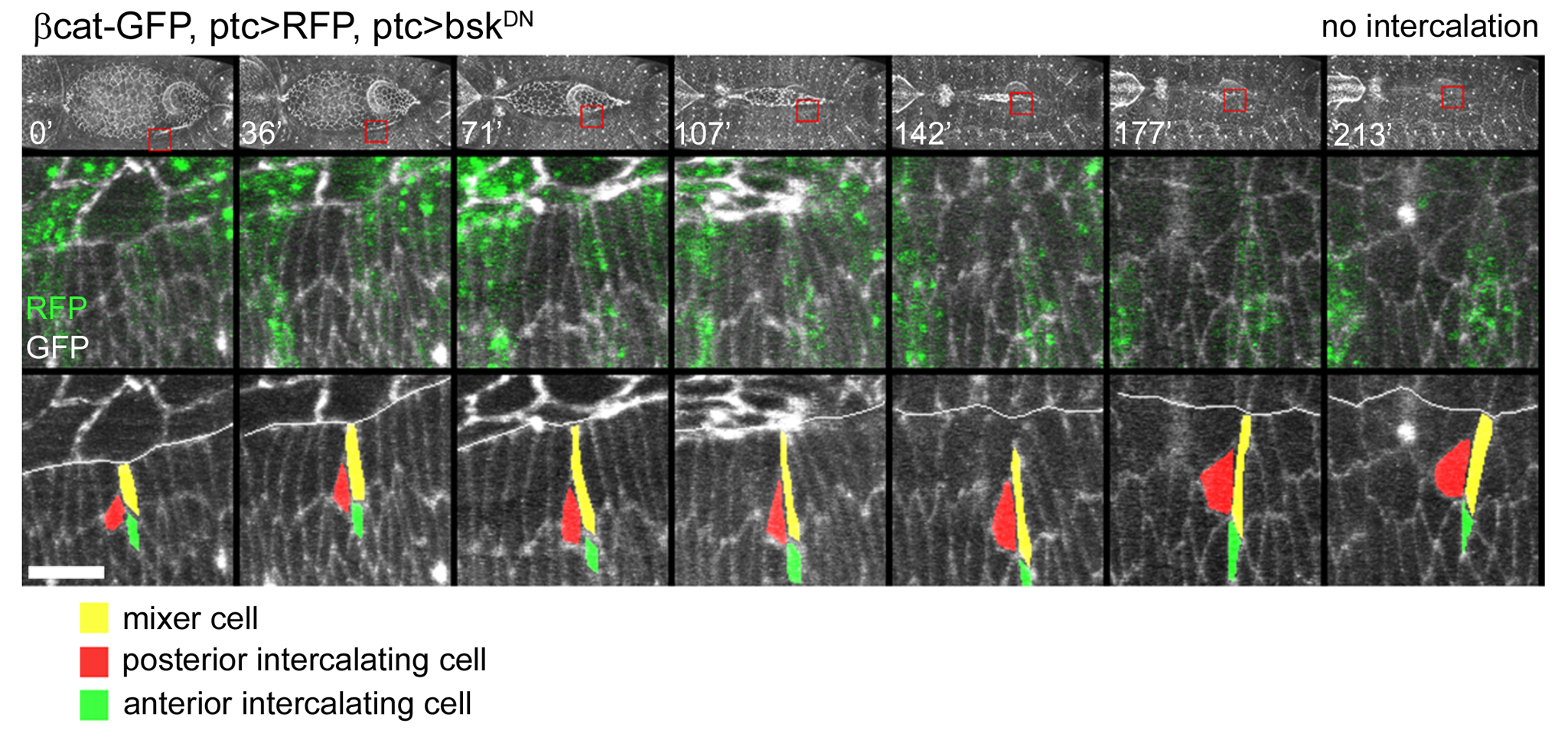

Supplement: Figure S4 — JNK down-regulation in the anterior compartment inhibits cell intercalation. Confocal still images from Video S4 showing an embryo expressing BskDN in the anterior compartment. Genotype: ptc-gal4, UAS-bskDN, UAS-RFP; βcatenin-GFP. βCatenin-GFP, white; RFP, green. Colour code as in Figure 1B. Scale bars: 10 µm. (2.31 MB TIF) [file pbio.1000390.s004.tif]

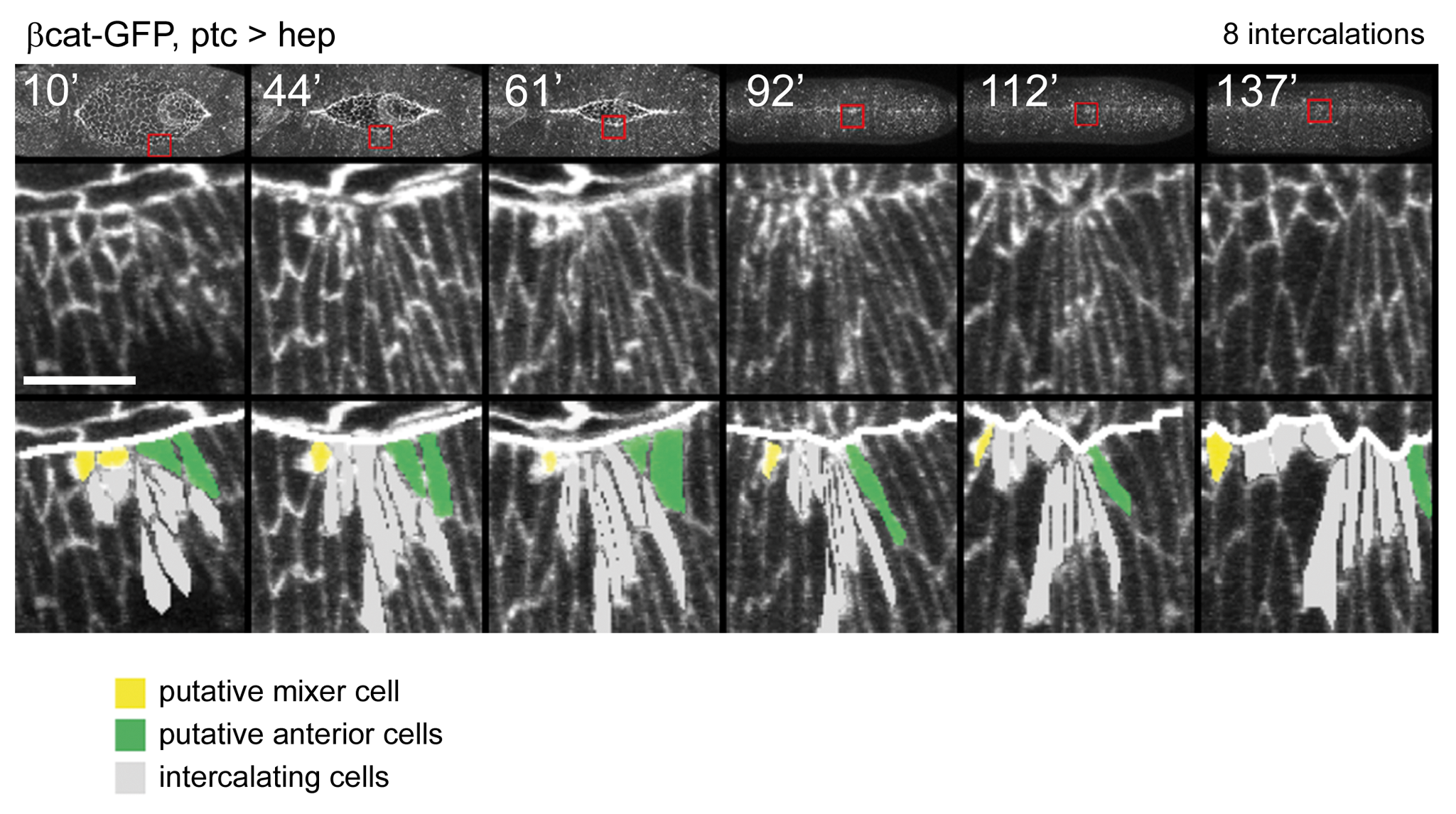

Supplement: Figure S5 — JNK overactivation induces ectopic mixer cells and intercalation. Confocal time-lapse imaging of an embryo expressing the JNKK Hep in the anterior compartment from Video S5. Genotype: ptc-gal4, UAS-hep; βcatenin-GFP. βCatenin-GFP, green. Colour code as in Figure 4A. Scale bars: 10 µm. (2.41 MB TIF) [file pbio.1000390.s005.tif]

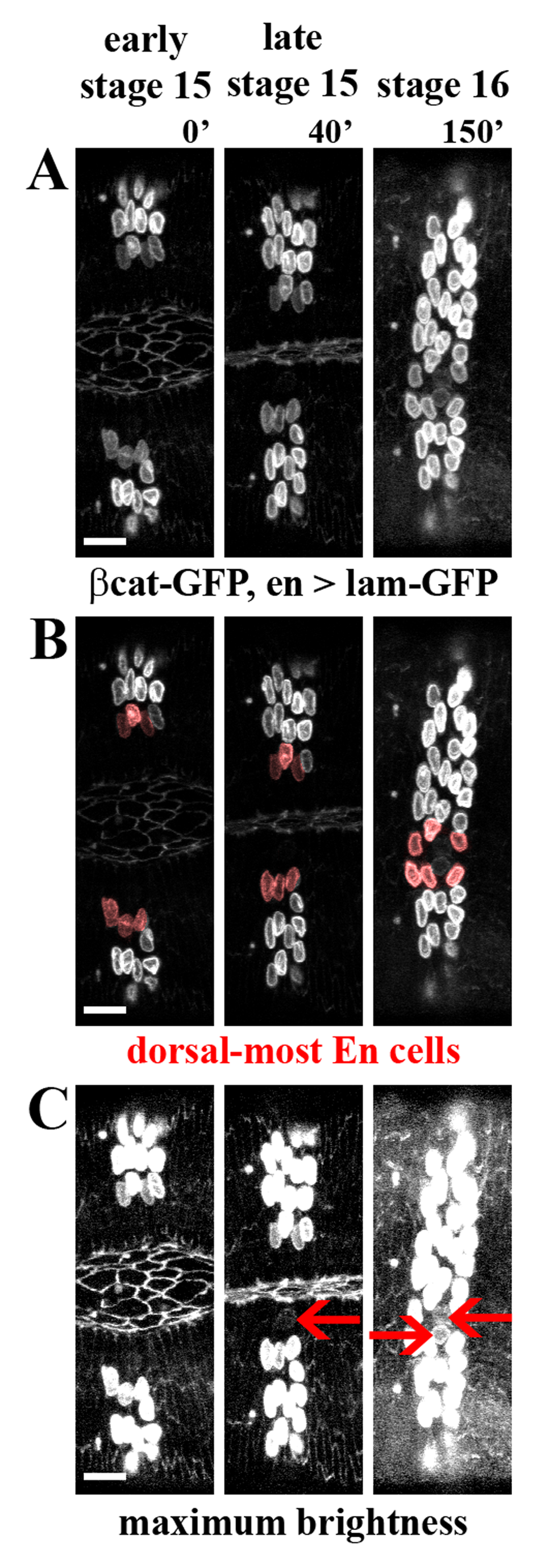

Supplement: Figure S6 — Expression pattern of the en-gal4 driver. Still images of an A4 segment of a βcatenin-GFP,en-gal4 > UAS-lamin-GFP live embryo, from early stage 15 to stage 16. (A) Normal image. (B) False colouring (red) of the dorsal row of En cells. (C) Increasing the brightness allows the visualization of lamin-GFP weak expression in the mixer cells (red arrows). Scale bars: 10 µm. (1.93 MB TIF) [file pbio.1000390.s006.tif]
