# Supplementary material for: JNK Signalling Controls Remodelling of the Segment Boundary through Cell Reprogramming during Drosophila Morphogenesis
Source: PLoS Biol. 2010 Jun 8;8(6):e1000390. doi: 10.1371/journal.pbio.1000390 (PMC2882433; doi:10.1371/journal.pbio.1000390)
Supplement: Table S1 — Timing and standard deviations of closure and cell intercalation described in Figure 1E . (0.04 MB DOC) [file pbio.1000390.s007.doc]

**Table S1: Timing and standard deviations of closure and intercalation described in Figure 1E**

| intersegments | T1-T2 | T2-T3 | T3-A1 | A1-A2 | A2-A3 | A3-A4 | A4-A5 | A5-A6 | A6-A7 | A7-A8 |
| --- | --- | --- | --- | --- | --- | --- | --- | --- | --- | --- |
| mean for closure time (min.) | -1 | 18 | 34 | 50 | 70 | 67 | 46 | 23 | 0 | -29 |
| corresponding s.d. (min.) | 6 | 9 | 15 | 22 | 19 | 9 | 5 | 9 |  | 5 |
| n (number of embryos) | 5 | 5 | 5 | 5 | 5 | 5 | 5 | 5 | 5 | 5 |
| mean for beginning of anterior intercalation (min.)  (the cell begins to elongate towards the LE) |  |  | 20 | -7 | -31 | -55 | -74 | -53 | -28 |  |
| corresponding s.d. (min.) |  |  |  | 37 | 22 | 33 | 14 | 22 | 10 |  |
| mean for end of anterior intercalation (min.)  (the cell is intercalated and part of the LE) |  |  | 106 | 60 | 33 | 36 | 0 | 33 | 50 |  |
| corresponding s.d. (min.) |  |  |  | 34 | 29 | 48 | 37 | 23 | 10 |  |
| n (number of intercalated cells) | 0 | 0 | 1 | 8 | 6 | 8 | 5 | 8 | 3 | 0 |
| mean for beginning of posterior intercalation (min.)  (the cell begins to elongate towards the LE) | -4 | 34 | 13 | -8 | -20 | -49 | -47 | -42 | -19 |  |
| corresponding s.d. (min.) | 5 | 12 | 49 | 28 | 29 | 30 | 24 | 33 | 18 |  |
| mean for end of posterior intercalation (min.)  (the cell is intercalated and part of the LE) | 52 | 91 | 74 | 56 | 37 | 16 | 24 | 20 | 34 |  |
| corresponding s.d. (min.) |  | 4 | 37 | 29 | 34 | 19 | 29 | 21 | 27 |  |
| n (number of posterior intercalated cells on the 11 edges) | 2 | 4 | 7 | 11 | 12 | 11 | 12 | 13 | 3 | 0 |
